# Second to fourth digit ratio (2D:4D) and concentrations of circulating sex hormones in adulthood

**DOI:** 10.1186/1477-7827-9-57

**Published:** 2011-04-27

**Authors:** David C Muller, Graham G Giles, Julie Bassett, Howard A Morris, John T Manning, John L Hopper, Dallas R English, Gianluca Severi

**Affiliations:** 1 Cancer Epidemiology Centre, The Cancer Council of Victoria, Melbourne, Australia; 2 Centre for Molecular, Environmental, Genetic, and Analytic Epidemiology, The University of Melbourne, Australia; 3 Hanson Institute, Institute of Medical and Veterinary Science, Adelaide, Australia; 4 MRC Epidemiology Research Centre, University of Southampton, Southampton General Hospital, UK

## Abstract

**Background:**

The second to fourth digit ratio (2D:4D) is used as a marker of prenatal sex hormone exposure. The objective of this study was to examine whether circulating concentrations of sex hormones and SHBG measured in adulthood was associated with 2D:4D.

**Methods:**

This analysis was based on a random sample from the Melbourne Collaborative Cohort Study. The sample consisted of of 1036 men and 620 post-menopausal women aged between 39 and 70 at the time of blood draw. Concentrations of circulating sex hormones were measured from plasma collected at baseline (1990-1994), while digit length was measured from hand photocopies taken during a recent follow-up (2003-2009). The outcome measures were circulating concentrations of testosterone, oestradiol, dehydroepiandrosterone sulphate, androstenedione, Sex Hormone Binding Globulin, androstenediol glucoronide for men only and oestrone sulphate for women only. Free testosterone and oestradiol were estimated using standard formulae derived empirically. Predicted geometric mean hormone concentrations (for tertiles of 2D:4D) and conditional correlation coefficients (for continuous 2D:4D) were obtained using mixed effects linear regression models.

**Results:**

No strong associations were observed between 2D:4D measures and circulating concentrations of hormones for men or women. For males, right 2D:4D was weakly inversely associated with circulating testosterone (predicted geometric mean testosterone was 15.9 and 15.0 nmol/L for the lowest and highest tertiles of male right 2D:4D respectively (*P*-*trend *= 0.04). There was a similar weak association between male right 2D:4D and the ratio of testosterone to oestradiol. These associations were not evident in analyses of continuous 2D:4D.

**Conclusions:**

There were no strong associations between any adult circulating concentration of sex hormone or SHGB and 2D:4D. These results contribute to the growing body of evidence indicating that 2D:4D is unrelated to adult sex hormone concentrations.

## Background

The length of the index finger divided by the length of the ring finger (2D:4D) has been proposed as a marker of prenatal androgen action. The investigation of digit ratios as possible markers of androgen action in early life began with the long-recognised observation that, compared with women, adult males tend to have longer ring fingers relative to other fingers. Contemporary anthropological studies have found small but consistent sex differences in 2D:4D, with men having lower average 2D:4D than women [1-3]. The ratio 2D:4D varies substantially by ethnicity, with more variability in 2D:4D accounted for by ethnic differences than sex differences [4]. However, the magnitude of sex differences in 2D:4D is similar across ethnic groups [4].

Studies have investigated possible associations between 2D:4D and a broad range of outcomes, including sexual orientation [5-11], sporting ability and physical fitness [12-18], and language development [19]. Sex hormone exposure in early life has also been implicated in the etiology of numerous diseases, including cancer [20].

There are several lines of evidence indicating that prenatal sex hormone exposure is associated with 2D:4D [2]. For instance the development of genitals and digits is controlled by the HoxA and HoxD genes [21]. Also, the sex difference in 2D:4D is observable at the end of the first trimester of fetal development [22]. Right hand 2D:4D measured at 2 years of age was found to be negatively correlated with the ratio of testosterone to oestradiol measured by amniocentesis in the second trimester [23], however this association was only observed for both sexes combined as the study was underpowered to detect within sex associations, and no strong association was observed between right 2D:4D and testosterone itself.

Perhaps the strongest evidence of the association between androgens and digit ratio comes from a consideration of links between 2D:4D and both the amount of testosterone before birth and an individual's sensitivity to testosterone. With regard to the former, children with congenital adrenal hyperplasia (CAH: a condition in which prenatal androgen levels are very high) have been shown to have lower mean 2D:4D than controls [3]. With regard to the latter, the CAG polymorphism in the androgen receptor influences sensitivity to androgens such that low CAGn is linked to high sensitivity. There is some evidence that low 2D:4D is associated with low CAGn [24], however a recent study has failed to replicate these findings [25]. Nevertheless, a strong link between 2D:4D and sensitivity to testosterone has been indicated by the recent finding that testosterone administration lowers cognitive empathy in women, but this effect is modified by 2D:4D such that participants with low 2D:4D show the greatest reduction in empathy [26]. There is both cross sectional [1] and longitudinal [27,28] evidence that sex differences in 2D:4D are unaffected by puberty, suggesting that 2D:4D is established very early in life. This makes 2D:4D an excellent candidate biomarker for examining putative associations between prenatal sex hormone exposure and risk of various sex hormone related conditions throughout the lifespan.

Typically any association between 2D:4D and an outcome *Y *is interpreted as a direct association between prenatal sex hormone exposure and *Y*. However this interpretation will be invalid if 2D:4D is also associated with adult hormone concentrations. In this case any association between 2D:4D and *Y *will not uniquely indicate an association with prenatal sex hormone exposure, but may also reflect an association between adult hormone concentrations and the outcome of interest. While the longitudinal stability of sex differences in 2D:4D suggests that a causal effect of adult hormone concentrations on 2D:4D can be excluded [27,28], it is widely assumed that prenatal and adult hormone concentrations are correlated. Consistent with this assumption, prenatal testosterone appears to stem from the fetus rather than the mother, as evidenced by the lack of association between concentrations of amniotic testosterone and maternal serum testosterone [29]. Therefore, since prenatal and adult sex hormones may stem from the same organs, their concentrations might be correlated. If there is a substantial correlation between prenatal and adult hormone concentrations we would expect to observe a correlation between 2D:4D measures and adult hormone concentrations.

Several studies to date have investigated possible associations between concentrations of adult sex hormones and 2D:4D, as well as the difference between right and left 2D:4D (Δ_*r*-*l*_) [1,5,30-37]. Each of these 10 studies, plus data from two new samples was included in a meta-analytic review of adult sex hormone concentrations and 2D:4D [38]. This meta-analysis of 332 women and 850 men aggregated correlations between adult hormone concentrations (measured from either serum or saliva) and 2D:4D, and overall found no association between 2D:4D and adult sex hormone concentrations, except in analyses that included a clinical sample of men attending a fertility clinic. We aim to examine whether 2D:4D or Δ_*r*-*l *_was associated with circulating hormone concentrations measured in a large, ethnically diverse sample of men and post-menopausal women participating in the Melbourne Collaborative Cohort Study (MCCS).

## Methods

### Study sample

The MCCS is a prospective cohort study of 41,514 people (17,045 men and 24,469 women) who were aged between 27 and 81 years at baseline (99.3% of whom were aged 40-69). Recruitment occurred between 1990 and 1994 in the Melbourne metropolitan area. Participants were recruited via the Electoral Rolls (enrollment to vote is compulsory for adults in Australia). Italian and Greek immigrants were deliberately over-sampled in order to include a broader range of diets and lifestyles in the study. There are 5,411 Italian and 4,525 Greek born participants, making up approximately 24% of the study sample. At baseline, participants were interviewed about their personal medical history and family history of disease, smoking and alcohol consumption, physical activity, and other social and lifestyle factors. A full reproductive history was sought from women, and all participants completed a food frequency questionnaire specifically developed for the MCCS. Physical measurements were also taken, and each participant provided a blood sample. Full details of the MCCS baseline phase are published elsewhere [39]. All participants provided written informed consent, and the study was approved by the Ethical Review Board at the Cancer Council Victoria.

Eligibility for the present study was restricted to a sample of 4659 MCCS participants (2167 men and 2492 women) who were selected for studies of plasma hormone concentrations and cancer [40,41]. The sample was chosen randomly from participants who had not been diagnosed with prostate, colorectal, breast or ovarian cancer prior to baseline and, for women only, were not using hormone replacement therapy (HRT) at baseline.

### Measurement of 2D:4D

During a face-to-face follow-up conducted during 2003-2009, participants had their hands photocopied for the purpose of measuring 2D:4D ratios. The length of the second (index) and fourth (ring) fingers were measured from photocopies of the surface of the hand using vernier calipers with a resolution of 0.01mm. Measurements were taken from the tip of the finger to the basal crease. Where two creases were visible at the base of the digit, the crease proximal to the palm was chosen. The length of the index finger was divided by the length of the ring finger to obtain 2D:4D, and Δ_*r*-*l *_was calculated as the difference between right and left 2D:4D. The measurement was undertaken by a team of trained research assistants at the Cancer Council Victoria.

### Measurement of circulating hormones

The methods used to measure circulating concentrations of sex hormone concentrations are described in detail in Baglietto et al. and Severi et al. [40,41].

Plasma samples were retrieved from liquid nitrogen, aliquoted into 450 μL volumes, and shipped on dry ice in batches of 80 samples to the laboratory where sex hormone binding globulin (SHBG), testosterone, total oestradiol, dehydroepiandrosterone sulphate (DHEAS), and androstenedione were measured for all participants. In addition, androstenediol glucoronide was measured for men and oestrone sulphate was measured for women. Assignment to batches was done randomly for each sex, and one scientist did all measurements. Ten percent of the samples in each batch were aliquots from pooled plasma that had been stored with the samples from participants.

Testosterone followed by total oestradiol was measured by electrochemiluminescence immunoas- say (Elecsys 2010 analyzer, Roche Diagnostics GmbH, Mannheim, Germany). DHEAS was measured by competitive immunoassay (IMMULITE analyzer, DPC, Los Angeles, CA). Androstenedione and androstenediol glucuronide were analysed by RIA (DSL-4200; TX). Oestrone sulphate was measured by RIA (DSL-5400). SHBG was measured by immunometric assay (IM- MULITE analyzer, DPC).

From the pooled plasma, the overall coefficients of variation were 7% (4% within batches and 5% between batches) for testosterone at a concentration of 4.3 nmol/L; 10% (8% within batches and 6% between batches) for oestradiol at a concentration of 157 pmol/L; 10% (9% within batches and 6% between batches) for DHEAS at a concentration of 4.0 μmol/L; 15% (11% within batches and 9% between batches) for androstenedione at a concentration of 2.6 nmol/L; 15% (13% within batches and 8% between batches) for oestrone sulphate at a concentration of 2.0 ng/mL; 10% (9% within batches and 5% between batches) for androstenediol glucoronide at a concentration of 8.0 ng/mL; and 7% (6% within batches and 4% between batches) for SHBG at a concentration of 45 nmol/L.

We examined the distribution of each sex hormone for implausible values, and excluded two men from analyses of testosterone (concentration < 1 nmol/L). No men or women had implausible values for any other hormone.

Circulating concentrations of free testosterone were calculated using the empirically developed formulae reported in [42]. Concentration of free oestradiol was calculated from the total concentration and from the concentration of SHBG using the law of mass action [43], under the assumption of a fixed albumin concentration of 40 g/L.

### Statistical analysis

One-hundred photocopies were measured by each research assistant twice so as to assess inter-and intra-observer reliability of the digit measurements. We fit mixed models to the raw digit lengths and 2D:4D ratios, with individual and research assistant entered into the model as crossed random effects. Intra-class correlation coefficients (ICC's) were calculated as the proportion of variance in digit length or digit ratio accounted for by between individual variation.

Linear regression was used to estimate mean differences in 2D:4D and Δ_*r*-*l *_by sex, and to estimate correlations between 2D:4D measures and age. Analysis of sex hormone concentrations and 2D:4D was undertaken separately for men and women. Using mixed-effects linear regression models fitted by maximum likelihood, natural log transformed values of sex hormone concentrations were regressed on sex-specific 2D:4D tertiles, and predicted geometric means and 95% confidence intervals (CI) were calculated for each tertile. The geometric mean can be considered an approximation of the median of untransformed hormone concentrations. The likelihood ratio test was used to test for trend across tertiles of 2D:4D. The ratios of testosterone to oestradiol and free testosterone to free oestradiol were also regressed on 2D:4D tertiles. Correlations between log hormone concentrations and continuous 2D:4D were obtained by standardising the coefficients from mixed-effects linear regression models. All models were adjusted for ethnicity, age at blood draw, BMI, and smoking status, and laboratory batch was entered as a random intercept. For females the models were also adjusted for previous oral contraceptive and HRT use, parity, duration of lactation, and age at menarche.

All analyses were performed using Stata/SE 11.1 for Linux 32 bit (Stata Corporation, College Station, TX).

## Results

Inter- and intra-observer reliability was very high for raw digit measurements, with ICC's for left and right, index and ring fingers all being greater than 0.95. ICC's for 2D:4D ratio were slightly lower than those for raw digit measurements (0.80 for right and 0.73 for left 2D:4D), but still suggest that the observed variability in digit ratio is largely due to individual differences rather than measurement error.

There were 408 men who had insufficient plasma, or whose results were deemed innappropriate for statistical analysis by the laboratory. A further 723 males did not have their hands photocopied, leaving 1036 men (48% of 2167 eligible) available for analysis. Of the 2492 eligible women, the laboratory returned 1875 results deemed appropriate for analysis. 714 women did not have their hands photocopied, and a further 529 women were classified as pre-menopausal at time of blood draw, rendering them ineligible for analysis. An additional 12 women who had missing values on potential confounders were excluded, leaving 620 women (42% of those post-menopausal women originally eligible) included in the present study.

The mean age at baseline was 54 for men and 60 for women. Seventy-nine percent of men and 80% of women were born in Australia, New Zealand, or the United Kingdom. The demographic, personal, and lifestyle characteristics of participants as well as mean plasma concentrations of hormones and 2D:4D are presented in Table 1 (men) and Table 2 (women).

**Table 1:** Demographic and lifestyle factors, and plasma concentrations of hormones and SHBG for the 1036 men

Characteristic	Category	Values
Age in years, mean (SD)		54 (9)
Country of birth, n (%)	Australia/New Zealand/UK/Other	816 (79)
	Italy	129 (12)
	Greece	91 (9)
Smoking, n (%)	Never	459 (44)
	Current	128 (12)
	Former	449 (43)
Alcohol consumption, n (%)	Abstainers	126 (12)
	Ex drinkers	49 (5)
	Low	693 (67)
	Medium	97 (9)
	High	71 (7)
BMI (kg/m2), mean (SD)		26.9 (3.4)
Right 2D:4D, mean (SD)	Overall	0.946 (0.036)
	Australia/New Zealand/UK/Other	0.948 (0.035)
	Italy	0.938 (0.041)
	Greece	0.941 (0.035)
Left 2D:4D, mean (SD)	Overall	0.954 (0.035)
	Australia/New Zealand/UK/Other	0.955 (0.035)
	Italy	0.949 (0.039)
	Greece	0.955 (0.032)
Δ_*r*-*l *_2D:4D, mean (SD)	Overall	-0.008 (0.033)
	Australia/New Zealand/UK/Other	-0.007 (0.031)
	Italy	-0.011 (0.041)
	Greece	-0.014 (0.034)
**Hormones and SHBG, geometric mean (interquartile range)^1^**		
Total testosterone (nmol/L)		15.5 (12.3,19.5)
Free testosterone (pmol/L)		244.1 (189.3,306.1)
Total oestradiol (pmol/L)		103.0 (87.0,122.0)
Free oestradiol (pmol/L)		1.7 (1.4,2.0)
DHEAS (μmol/L)		3.5 (2.3,5.2)
Androstenedione (nmol/L)		4.1 (3.2,5.4)
Androstenediol glucoronide (ng/mL)		14.8 (10.3,20.2)
SHBG (nmol/L)		35.2 (27.0,43.8)
Testosterone:oestradiol ratio		151.6 (118.5,187.3)

^1^At baseline blood collection. Number of missing measures were 2 for testosterone, 2 for free testosterone,and 66 for androstenediol glucoronide.

**Table 2:** Demographic and lifestyle factors, and plasma concentrations of hormones and SHBG for the 620 women

Characteristic	Category	Values
Age in years, mean (SD)		60 (6)
Age at menopause in years, mean (SD)		50 (4)
Country of birth, n (%)	Australia/New Zealand/UK/Other	496 (80)
	Italy	83 (13)
	Greece	41 (7)
Smoking, n (%)	Never	446 (72)
	Current	42 (7)
	Former	132 (21)
Alcohol consumption, n (%)	Abstainers	267 (43)
	Ex drinkers	16 (3)
	Low	268 (43)
	Medium	57 (9)
	High	12 (2)
Age at menarche, n (%)	<12	104 (17)
	12	126 (20)
	13	149 (24)
	>= 14	241 (39)
Parity, n (%)	Nulliparous	72 (12)
	<25 years and 1 pregnancy	38 (6)
	<25 years and >1 pregnancy	175 (28)
	>= 25 years and 1 pregnancy	169 (27)
	>= 25 years and >1 pregnancy	166 (27)
Duration of lactation, n (%)	never	156 (25)
	<= 6 months	123 (20)
	7-12 months	104 (17)
	13-24 months	141 (23)
	> 24 months	96 (15)
Oral Contraceptive use, n (%)	Never	326 (53)
	Ex	294 (47)
HRT use, n (%)	Never	531 (86)
	Ex	89 (14)
BMI (kg/m2), mean (SD)		27.3 (4.9)
Right 2D:4D, mean (SD)	Overall	0.953 (0.035)
	Australia/New Zealand/UK/Other	0.954 (0.035)
	Italy	0.954 (0.034)
	Greece	0.945 (0.030)
Left 2D:4D, mean (SD)	Overall	0.961 (0.036)
	Australia/New Zealand/UK/Other	0.961 (0.036)
	Italy	0.963 (0.036)
	Greece	0.962 (0.038)
Δ_*r*-*l *_2D:4D, mean (SD)	Overall	-0.008 (0.035)
	Australia/New Zealand/UK/Other	-0.007 (0.035)
	Italy	-0.010 (0.035)
	Greece	-0.017 (0.035)
**Hormones and SHBG, geometric mean (interquartile range)^1^**		
Total testosterone (nmol/L)		0.7 (0.4,1.1)
Free testosterone (pmol/L)		7.5 (3.5,12.5)
Total oestradiol (pmol/L)		57.0 (45.0,73.0)
Free oestradiol (pmol/L)		0.8 (0.6,1.1)
DHEAS (μmol/L)		1.5 (0.9,2.5)
Androstenedione (nmol/L)		2.5 (1.6,3.5)
Oestrone sulphate (ng/mL)		1.0 (0.7,1.4)
SHBG (nmol/L)		50.4 (37.7,68.3)
Testosterone:oestradiol ratio		12.5 (7.8,18.4)

^1^At baseline blood collection. Number of missing measures were 54 for free testosterone and 13 for oestrone sulphate.

Based on analyses of the whole random sample (n = 2575), women had higher mean 2D:4D than men for both left and right hands. The mean differences in the ratios were 0.014 (95% CI, 0.011 - 0.016; Cohen's *d *= 0.37) for the right hand, and 0.013 (95% CI, 0.010 - 0.015; Cohen's *d *= 0.35) for the left hand. The estimated sex differences were not changed materially by adjusting for age and ethnicity. There were no substantial sex differences in Δ_*r*-*l*_, though it was slightly higher for women than men on average (mean difference of 0.001; 95% CI, -0.002 - 0.003). Greek and Italian participants had slightly lower 2D:4D than Anglo-Celtic participants, but these differences were insubstantial (all mean differences < 0.009). Greek and Italian participants also had lower Δ_*r*-*l *_than Anglo-Celtic participants (Tables 1 and 2). There were small reductions in 2D:4D with increasing age (for example, 0.009 (95% CI, 0.0007 - 0.010) reduction in right 2D:4D for each 10 year increase in age). The unadjusted pairwise correlation between right 2D:4D and age was -0.23 (*P *< 0.001) for men and -0.18 (*P *< 0.001) for women. Similarly, for left 2D:4D the correlation coefficient was -0.15 (*P *< 0.001) for men and -0.14 for women (*P *< 0.001). Undadjusted pairwise correlations between Δ_*r*-*l *_and age were -0.09 (*P *= 0.002) for men and -0.05 (*P *= 0.08) for women.

Tables 3 and 4 show the predicted (by mixed effects linear regression models) geometric mean concentration for each hormone and SHBG by tertiles of right and left 2D:4D and Δ_*r*-*l *_for men and women respectively. There was a modest inverse association between 2D:4D for the right hand and plasma testosterone concentration for men. Mean testosterone concentration was 15.9, 15.3, and 15.0 nmol/L for tertiles I, II, and III of right 2D:4D respectively (likelihood ratio test for trend *P *= 0.04). Left 2D:4D and Δ_*r*-*l *_were not associated with circulating concentrations of total or free testosterone in males. We observed extremely small positive associations between Δ_*r*-*l *_and both total and free testosterone concentrations for women. Mean testosterone concentration was 0.6, 0.7, and 0.7 nmol/L (likelihood ratio test for trend *P *= 0.04), and mean free testosterone concentrations were 5.2, 6.9, and 6.5 pmol/L (likelihood ratio test for trend *P *= 0.05) for tertiles I, II, and III of Δ_*r*-*l *_respectively. No other association between left or right 2D:4D or Δ_*r*-*l *_and any circulating hormone or SHBG was evident for men or women.

**Table 3:** Predicted geometric mean (G^1^) hormone concentration in men by tertiles of right and left 2D:4D and Δ_*r*-*l*_.

		**Right Hand**					**Left Hand**					**Δ_*r*-*l*_**				
			
**2D:4D tertile**		**G**	**(95% CI)**	***P^2^_trend_***	***ρ^3^***	***P^4^***	**G**	**(95% CI)**	***P^2^_trend_***	***ρ^3^***	***P^4^***	**G**	**(95% CI)**	***P^2^_trend_***	***ρ^3^***	***P^4^***
			
Testosterone (nmol/l)	I	15.9	(15.3 - 16.4)	0.04	-0.05	0.12	15.6	(15.0 - 16.2)	0.34	-0.03	0.34	15.5	(14.9 - 16.0)	0.86	-0.02	0.44
	II	15.3	(14.8 - 15.9)				15.3	(14.7 - 15.8)				15.3	(14.7 - 15.8)			
	III	15.0	(14.5 - 15.6)				15.2	(14.7 - 15.8)				15.4	(14.9 - 16.0)			
Free testosterone (pmol/l)	I	242.1	(232.1 - 252.0)	0.07	-0.04	0.19	237.4	(227.7 - 247.2)	0.50	-0.02	0.51	236.6	(226.8 - 246.4)	0.81	-0.03	0.42
	II	233.5	(224.0 - 243.0)				232.8	(223.3 - 242.3)				232.8	(223.2 - 242.4)			
	III	229.1	(219.7 - 238.6)				232.7	(223.1 - 242.3)				234.9	(225.2 - 244.6)			
Oestradiol (pmol/l)	I	104.3	(99.6 - 108.9)	0.73	0.02	0.45	104.5	(99.8 - 109.2)	0.83	0.02	0.41	104.4	(99.8 - 109.1)	0.84	-0.00	0.96
	II	105.6	(100.9 - 110.3)				105.3	(100.6 - 109.9)				105.7	(101.0 - 110.5)			
	III	105.0	(100.3 - 109.7)				104.9	(100.3 - 109.6)				104.9	(100.2 - 109.6)			
Free oestradiol (pmol/l)	I	1.7	(1.6 - 1.8)	0.51	0.03	0.31	1.7	(1.6 - 1.8)	0.58	0.03	0.26	1.7	(1.6 - 1.8)	0.92	-0.00	0.99
	II	1.7	(1.7 - 1.8)				1.7	(1.6 - 1.8)				1.7	(1.7 - 1.8)			
	III	1.7	(1.6 - 1.8)				1.7	(1.6 - 1.8)				1.7	(1.6 - 1.8)			
DHEAS (μmol/l)	I	3.3	(3.1 - 3.5)	0.37	-0.03	0.28	3.4	(3.2 - 3.6)	0.07	-0.04	0.13	3.3	(3.1 - 3.5)	0.68	0.01	0.64
	II	3.4	(3.2 - 3.6)				3.2	(3.0 - 3.4)				3.2	(3.0 - 3.4)			
	III	3.2	(3.0 - 3.4)				3.2	(3.0 - 3.3)				3.4	(3.2 - 3.6)			
Androstenedione (nmol/l)	I	4.2	(3.9 - 4.4)	0.30	-0.05	0.12	4.2	(4.0 - 4.4)	0.31	-0.04	0.26	4.2	(4.0 - 4.4)	0.96	-0.02	0.52
	II	4.2	(3.9 - 4.4)				4.2	(3.9 - 4.4)				4.0	(3.8 - 4.2)			
	III	4.0	(3.8 - 4.2)				4.0	(3.8 - 4.2)				4.2	(4.0 - 4.4)			
Androstenediol glucoronide (ng/ml)	I	13.9	(13.0 - 14.8)	0.11	0.05	0.11	14.0	(13.2 - 14.9)	0.32	0.03	0.32	14.2	(13.3 - 15.1)	0.49	0.02	0.57
	II	14.3	(13.4 - 15.2)				14.4	(13.5 - 15.3)				14.2	(13.4 - 15.1)			
	III	14.8	(13.9 - 15.7)				14.6	(13.7 - 15.5)				14.6	(13.7 - 15.5)			
SHHB (nmol/l)	I	35.0	(33.6 - 36.3)	0.50	-0.02	0.57	34.7	(33.4 - 36.0)	0.60	-0.02	0.60	34.5	(33.2 - 35.8)	0.98	-0.01	0.82
	II	33.9	(32.6 - 35.2)				34.0	(32.8 - 35.3)				34.0	(32.7 - 35.3)			
	III	34.3	(33.0 - 35.6)				34.2	(33.0 - 35.5)				34.5	(33.2 - 35.8)			

^1^Estimates from linear mixed models of the logarithm of hormone concentration on 2D:4D tertiles with laboratory batch entered as a random intercept, adjusted for ethnicity, age at blood draw, BMI, and smoking status.

^2^From likelihood ratio test for linear trend across 2D:4D tertiles.

^3^Standardised regression coefficients (correlations) from linear mixed models of the logarithm of hormone concentration on continuous 2D:4D with laboratory batch entered as a random intercept, adjusted for ethnicity, age at blood draw, BMI, and smoking status.

^4^From likelihood ratio test of *ρ*.

**Table 4:** Predicted geometric mean (G^1^) hormone concentration in women by tertiles of right and left 2D:4D and Δ_*r*-*l*_.

		**Right Hand**					**Left Hand**					**Δ_*r*-*l*_**				
			
**2D:4D tertile**		**G**	**(95% CI)**	***P^2^_trend_***	***ρ^3^***	***P^4^***	**G**	**(95% CI)**	***P^2^_trend_***	***ρ^3^***	***P^4^***	**G**	**(95% CI)**	***P^2^_trend_***	***ρ^3^***	***P^4^***
			
Testosterone (nmol/L)	I	0.7	(0.6 - 0.8)	0.76	-0.01	0.74	0.7	(0.6 - 0.8)	0.10	-0.03	0.41	0.6	(0.5 - 0.7)	0.04	0.02	0.55
	II	0.7	(0.6 - 0.7)				0.7	(0.6 - 0.7)				0.7	(0.6 - 0.8)			
	III	0.7	(0.6 - 0.7)				0.6	(0.5 - 0.7)				0.7	(0.6 - 0.8)			
Free Testosterone (pmol/L)	I	6.0	(4.9 - 7.1)	0.92	-0.03	0.49	6.5	(5.3 - 7.6)	0.16	-0.04	0.34	5.2	(4.3 - 6.1)	0.05	0.02	0.71
	II	6.6	(5.4 - 7.8)				6.4	(5.3 - 7.6)				6.9	(5.6 - 8.1)			
	III	5.9	(4.9 - 7.0)				5.6	(4.6 - 6.5)				6.5	(5.3 - 7.6)			
Oestradiol (pmol/L)	I	57.0	(52.1 - 61.9)	0.50	0.02	0.63	58.0	(52.9 - 63.0)	0.77	0.02	0.64	55.1	(50.3 - 59.9)	0.62	-0.00	0.91
	II	55.6	(50.8 - 60.3)				55.3	(50.5 - 60.1)				59.7	(54.5 - 64.8)			
	III	58.7	(53.7 - 63.7)				57.2	(52.2 - 62.2)				56.4	(51.5 - 61.3)			
Free oestradiol (pmol/L)	I	0.8	(0.7 - 0.9)	0.85	-0.00	0.89	0.8	(0.7 - 0.9)	0.64	0.00	0.95	0.8	(0.7 - 0.8)	0.73	-0.01	0.73
	II	0.8	(0.7 - 0.8)				0.8	(0.7 - 0.8)				0.8	(0.7 - 0.9)			
	III	0.8	(0.7 - 0.9)				0.8	(0.7 - 0.9)				0.8	(0.7 - 0.8)			
DHEAS (μmol/L)	I	1.5	(1.3 - 1.6)	0.38	-0.02	0.63	1.6	(1.4 - 1.7)	0.08	-0.02	0.62	1.4	(1.2 - 1.5)	0.44	0.00	0.91
	II	1.4	(1.3 - 1.6)				1.4	(1.3 - 1.5)				1.4	(1.3 - 1.6)			
	III	1.4	(1.2 - 1.5)				1.4	(1.2 - 1.5)				1.5	(1.3 - 1.6)			
Androstenedione (nmol/L)	I	2.4	(2.2 - 2.6)	0.26	-0.04	0.40	2.4	(2.2 - 2.7)	0.16	-0.05	0.27	2.2	(2.0 - 2.4)	0.10	0.01	0.77
	II	2.3	(2.1 - 2.5)				2.3	(2.1 - 2.5)				2.3	(2.1 - 2.5)			
	III	2.2	(2.1 - 2.4)				2.3	(2.1 - 2.4)				2.4	(2.2 - 2.6)			
Oestrone sulphate (ng/mL)	I	1.1	(1.0 - 1.2)	0.68	-0.03	0.44	1.1	(1.0 - 1.2)	0.35	-0.01	0.85	1.0	(0.9 - 1.1)	0.51	-0.01	0.72
	II	1.0	(0.9 - 1.1)				1.0	(0.9 - 1.1)				1.0	(0.9 - 1.1)			
	III	1.0	(1.0 - 1.1)				1.0	(0.9 - 1.1)				1.1	(1.0 - 1.1)			
SHGB (nmol/L)	I	48.3	(45.3 - 51.3)	0.26	0.06	0.13	49.5	(46.4 - 52.6)	0.74	0.04	0.26	50.0	(46.8 - 53.1)	0.65	0.02	0.66
	II	50.8	(47.7 - 54.0)				50.0	(46.9 - 53.0)				48.6	(45.6 - 51.6)			
	III	50.6	(47.5 - 53.8)				50.2	(47.1 - 53.3)				50.9	(47.8 - 54.1)			

^1^Estimates from linear mixed models of the logarithm of hormone concentration on 2D:4D tertiles with laboratory batch entered as a random intercept, adjusted for ethnicity, age at blood draw, BMI, smoking status, previous oral contraceptive and HRT use, parity, duration of lactation, and age at menarche.

^2^From likelihood ratio test for linear trend across 2D:4D tertiles.

^3^Standardised regression coefficients (correlations) from linear mixed models of the logarithm of hormone concentration on continuous 2D:4D with laboratory batch entered as a random intercept, adjusted for ethnicity, age at blood draw, BMI, smoking status, previous oral contraceptive and HRT use, parity, duration of lactation, and age at menarche.

^4^From likelihood ratio test of *ρ*.

Possible associations between the relative concentration of testosterone and oestradiol and 2D:4D ratios were also assessed (Tables 5 and 6). The concentration of circulating testosterone relative to oestradiol was decreased with increasing right 2D:4D for males - men in the lowest tertile of 2D:4D had a testosterone concentration 160 times greater than their oestradiol concentration (95% CI, 153 - 168), compared with men in the highest tertile of 2D:4D who had 152 times higher circulating testosterone relative to oestradiol (95% CI, 147 - 160); Ptrend = 0.03). A similar inverse association was seen between right 2D:4D and relative concentrations of free testosterone and free oestradiol, with men in the lowest 2D:4D tertile having 157 times higher circulating testosterone relative to oestradiol (95% CI 149 - 165) and men in the highest 2D:4D tertile having 146 times higher testosterone relative to oestradiol (95% CI 138 - 155; Ptrend = 0.02) There was no statistical evidence that left 2D:4D or Δ_*r*-*l *_were associated with relative concentration of testosterone and oestradiol for males. Neither right or left 2D:4D or Δ_*r*-*l *_were associated with relative concentrations of testosterone and oestradiol for females.

**Table 5:** Predicted mean^1 ^relative concentrations of testosterone and oestradiol in men by tertiles of right and left 2D:4D and Δ_*r*-*l*_.

		**Right Hand**					**Left Hand**					**Δ_*r*-*l*_**				
			
**2D:4D tertile**		**mean**	**(95% CI)**	***P^2^_trend_***	***ρ^3^***	***P^4^***	**mean**	**(95% CI)**	***P^2^_trend_***	***ρ^3^***	***P^4^***	**mean**	**(95% CI)**	***P^2^_trend_***	***ρ^3^***	***P^4^***
			
Testosterone:oestradiol	I	160.2	(152.6 - 167.9)	0.03	-0.06	0.03	158.9	(151.3 - 166.5)	0.19	-0.05	0.07	155.7	(148.1 - 163.4)	0.82	-0.01	0.69
	II	154.1	(146.5 - 161.8)				153.4	(145.9 - 160.9)				154.0	(146.4 - 161.7)			
	III	152.1	(144.5 - 159.8)				154.2	(146.6 - 161.7)				156.5	(148.9 - 164.2)			
Free testosterone:free oestradiol	I	157.0	(148.6 - 165.4)	0.02	-0.07	0.02	155.4	(147.1 - 163.8)	0.11	-0.06	0.04	151.1	(142.6 - 159.5)	0.92	-0.01	0.64
	II	148.5	(140.1 - 156.9)				147.4	(139.0 - 155.7)				148.3	(139.9 - 156.8)			
	III	146.2	(137.8 - 154.6)				148.1	(139.7 - 156.4)				151.5	(143.0 - 160.0)			

^1^Estimates from linear mixed models of the ratio of hormone concentrations on 2D:4D tertiles with laboratory batch entered as a random intercept, adjusted for ethnicity, age at blood draw, BMI, and smoking status.

^2^From likelihood ratio test for linear trend across 2D:4D tertiles.

^3^Standardised regression coefficients (correlations) from linear mixed models of the logarithm of hormone concentration on continuous 2D:4D with laboratory batch entered as a random intercept, adjusted for ethnicity, age at blood draw, BMI, and smoking status.

^4^From likelihood ratio test of *ρ*.

**Table 6:** Predicted mean^1 ^relative concentrations of testosterone and oestradiol in women by tertiles of right and left 2D:4D and Δ_*r*-*l*_.

		**Right Hand**					**Left Hand**					**Δ_*r*-*l*_**				
			
**2D:4D tertile**		**mean**	**(95% CI)**	***P^2^_trend_***	***ρ^3^***	***P^4^***	**mean**	**(95% CI)**	***P^2^_trend_***	***ρ^3^***	***P^4^***	**mean**	**(95% CI)**	***P^2^_trend_***	***ρ^3^***	***P^4^***
			
Testosterone:oestradiol	I	15.0	(13.0 - 16.9)	0.39	-0.03	0.38	14.6	(12.6 - 16.5)	0.56	-0.03	0.52	14.0	(12.1 - 16.0)	0.24	-0.00	0.98
	II	14.7	(12.8 - 16.6)				15.4	(13.4 - 17.3)				14.6	(12.6 - 16.5)			
	III	14.2	(12.3 - 16.1)				14.0	(12.1 - 16.0)				15.1	(13.2 - 17.1)			
Free testosterone:free oestradiol	I	11.4	(9.6 - 13.3)	0.58	-0.04	0.32	11.2	(9.4 - 13.0)	0.68	-0.02	0.60	10.7	(8.8 - 12.5)	0.31	-0.01	0.83
	II	11.6	(9.7 - 13.4)				11.9	(10.1 - 13.7)				11.4	(9.6 - 13.3)			
	III	10.9	(9.1 - 12.7)				10.8	(9.0 - 12.6)				11.7	(9.8 - 13.5)			

^1^Estimates from linear mixed models of the ratio of hormone concentrations on 2D:4D tertiles with laboratory batch entered as a random intercept, adjusted for ethnicity, age at blood draw, BMI, smoking status, previous oral contraceptive and HRT use, parity, duration of lactation, and age at menarche.

^2^From likelihood ratio test for linear trend across 2D:4D tertiles.

^3^Standardised regression coefficients (correlations) from linear mixed models of the logarithm of hormone concentration on continuous 2D:4D with laboratory batch entered as a random intercept, adjusted for ethnicity, age at blood draw, BMI, smoking status, previous oral contraceptive and HRT use, parity, duration of lactation, and age at menarche.

^4^From likelihood ratio test of *ρ*.

## Discussion

We found that although women had slightly higher average 2D:4D than men, there was substantial overlap of the distributions of 2D:4D for men and women. There were no notable differences in 2D:4D between participants of Anglo-Celtic, Italian, or Greek ethnic backgrounds, though Italian and Greek participants had slightly lower Δ_*r*-*l *_than participants of Anglo-Celtic descent. We found that older participants had slightly lower 2D:4D measures than younger participants on average. This association indicates that there may be birth cohort effects on the underlying determinants of 2D:4D.

No hormone or SHBG examined in the present study was strongly associated with either left or right 2D:4D for men or women. The weak inverse associations observed between right 2D:4D and total testosterone, as well as testosterone relative to oestradiol for men were all in the expected direction, with increasing 2D:4D associated with decreasing concentrations of testosterone and testosterone relative to oestradiol. The weak association between Δ_*r*-*l *_and testosterone for women was not in the expected direction. The estimated conditional correlations between 2D:4D measures and hormone concentrations were all close to zero, indicating that the detected associations are negligible.

Previous studies have suggested that the relative length of the 2nd and 4th digit (2D:4D) is related to prenatal sex hormones, with low 2D:4D associated with exposure to higher concentrations of prenatal testosterone relative to oestradiol. This has led to the use of 2D:4D as a tool to investigate possible associations between prenatal sex hormone exposure and various behaviours, diseases, and disorders. If 2D:4D is also associated with adult circulating hormone concentrations then inference in such studies becomes complicated, as any association between 2D:4D and an outcome will be conflated with adult hormone concentrations. In this case measuring 2D:4D would have limited added value when hormone concentrations have been measured directly.

Our results are generally consistent with those reported previously. The only studies to date that have reported moderate correlations between adult sex hormone concentrations and 2D:4D are analyses of samples of men attending fertility clinics [1,37]. Studies of non-clinical samples have typically reported null results, and a recent meta analytic review of all studies only reported significant correlations for analyses that included samples of patients from fertility clinics [38].

The finding that there is substantial overlap in the distributions of 2D:4D for males and females has been previously reported, and it has been suggested on this basis that 2D:4D can be rejected as a biomarker of prenatal androgen exposure [44,45]. It is certainly true that discrimination and prediction of an individual's phenotype (such as sex) based soley on 2D:4D is highly inaccurate. However there remains much evidence that prenatal androgens affect 2D:4D, and the distinction needs to be made between the use of 2D:4D as a discriminative or predictive tool, and 2D:4D as a (albeit imperfect) tool to study possible associations [46].

Our study extends the results of previous studies to an older population. The age range of participants in this study is also wider than that in any single previous study. Our study is by far the largest to date, with sufficient power to detect very small differences in sex hormone concentrations. Also, we were able to adjust our models for numerous factors that were collected during the baseline interview. The reproducibility of the hormone and digit measurements is also a strength of our study.

This study has some limitations. Blood samples were collected at various times throughout the day, so circadian rhythms in hormone secretion is a source of variance in hormone measurements that might make correlations difficult to detect. Also, blood samples were collected at recruitment into the cohort, whereas digit ratios were measured during routine follow up at least ten years later. Not all participants attended follow up, and even though it is unlikely that participants lost to follow up differ systematically from those who attended in terms of digit ratio, we cannot rule out the possibility that our sample is somehow biased. This is unlikely to be a problem for the interpretation of associations, as there is some evidence that 2D:4D remains stable over time [28]. Also, due to fluctuations in concentrations of circulating hormones throughout the menstrual cycle, we have not been able to assess whether 2D:4D is associated with circulating hormone concentrations for pre-menopausal women. Another limitation of our study is that we measured digits from photocopies of the hand rather than directly on the ventral surface of the hand. Photocopies have been shown to yield lower 2D:4D values than direct measurement [47]. This limitation is not critical, as our measurement technique was identical for all our participants.

## Conclusions

No circulating hormone or SHGB was strongly associated with 2D:4D for men or women. These results contribute to the growing body of evidence that 2D:4D is unrelated to circulating hormone concentrations in adulthood. To the extent that 2D:4D reflects sensitivity to and prenatal exposure to sex hormones, these results suggest that associations between 2D:4D and hormone related behaviours, diseases, and disorders can be interpreted directly, independent of any effects of adult circulating sex hormones.

## Competing interests

The authors declare that they have no competing interests.

## Authors' contributions

DCM planned and performed the statistical analysis and drafted the manuscript. JB independently verified results of the statistical analysis and critically revised the manuscript. HAM measured the hormone concentrations and critically revised the manuscript. JTM provided advice regarding the measurement of digit ratios and critically revised the manuscript. GGG, JLH, DRE, and GS conceived the study, participated in the planning of the statistical analysis, and critically revised the manuscript. All authors read and approved the final manuscript.
